# The effect of hybrid ultrasound/fluoroscopy guidance vs only fluoroscopy guidance on procedure time and radiation exposure in caudal epidural steroid injections^☆^

**DOI:** 10.1016/j.inpm.2025.100567

**Published:** 2025-03-05

**Authors:** Serdar Kesikburun, Şahide Eda Artuç, Esra Çelik Karbancioğlu, Bilge Kesikburun, Emre Adigüzel, Evren Yaşar

**Affiliations:** a Department of Physical Medicine and Rehabilitation, University of Health Sciences, Gulhane Medical School, Ankara, Türkiye; b Department of Physical Medicine and Rehabilitation, University of Health Sciences, Ankara Gaziler Physical Therapy and Rehabilitation Training and Research Hospital, Ankara, Türkiye; c Department of Physical Medicine and Rehabilitation, University of Health Sciences, Ankara City Hospital, Ankara, Türkiye

**Keywords:** Caudal epidural injections, Radiation exposure, Ultrasonography, Fluoroscopy, Interventional pain management

## Abstract

**Background:**

Ultrasonography guidance have had a rapid increase in its popularity for caudal epidural steroid injections. However, unlike fluoroscopy, ultrasound cannot reliably detect intravascular or intradural distribution of the medication. Therefore, the practitioners cannot be entirely certain about the accuracy of the procedure. A hybrid technique may eliminate these drawbacks. The primary objective of this study is to evaluate the effect of hybrid ultrasound/fluoroscopy guidance on procedure time, fluoroscopy duration, and radiation exposure during caudal epidural injections, compared to the conventional method of fluoroscopy-only guidance.

**Methods:**

In this prospective randomized controlled trial, 65 patients who were undergoing caudal epidural steroid injection randomized into two groups: the hybrid ultrasound/fluoroscopy group [Group 1 (n = 32)] and the fluoroscopy-only group [Group 2 (n = 33)]. Kerma area product (KAP), elapsed time of the needle insertion into sacral hiatus, elapsed time of the entire procedure and fluoroscopy time were measured. Numeric Rating Scale (NRS) for pain level and Oswestry Disability Index (ODI) were also assessed before the procedure and two weeks later.

**Results:**

Radiation exposure measured using fluoroscopy time (group 1 = 0.06 ± 0.01 min; group 2 = 0.09 ± 0.03 min) and KAP (group 1 = 43.73 ± 16.90 cGy cm^2^; group 2 = 72.39 ± 32.75 cGy cm^2^) was significantly lower in group 1 compared to group 2 (p < 0.001 for both). Elapsed time of the needle insertion into sacral hiatus (T1) (group 1 = 2.82 ± 1.07 min; group 2 = 3.73 ± 2.47) was shorter in the group 1 compared to group 2 (p = 0.027). However, there was no significant difference in the entire procedure time (group 1 = 5.14 ± 1.55 min; group 2 = 5.86 ± 2.71 min) between group 1 and group 2 (p = 0.100). A significant improvement in NRS and ODI measurements was shown over time for both groups (p < 0.001 for both). No significant interaction between group and time was identified concerning NRS (p = 0.177) and ODI (p = 0.207) scores. A total of 4 vascular uptake out of 65 procedures (6.1 %) were detected in both groups.

**Conclusions:**

The hybrid guidance may offer a potentially safer method minimizing radiation risk compared to fluoroscopy-only guidance for caudal epidural steroid injections.

## 1 Introduction

Epidural injections are frequently used interventional pain treatments for various conditions that cause low back pain, such as lumbar disc herniation-radiculopathy, spinal stenosis, post-lumbar laminectomy syndrome, and intervertebral disc disease. The epidural space can be accessed through three different approaches: “caudal,” “transforaminal,” and “interlaminar.” In the caudal approach, the needle is inserted into the sacral canal through the sacral hiatus [1,2]. While being less selective compared to the transforaminal approach, the caudal epidural steroid injection (ESI) is considered safer in terms of avoiding dural puncture and subsequent spinal headaches [3]. In patients with a history of spinal surgery, the caudal approach provides technical ease for epidural injection due to scar tissue in the lumbar region [4]. The presence of the sacral venous plexus which might be risk for intravascular injection and variations observed in the sacral hiatus constitute the technical disadvantages of the caudal approach.

It has been shown that if these interventional procedures are performed without the use of imaging techniques, there is a high injection error rate (20–38 %) and the treatment will be ineffective [5]. While ionizing radiation-emitting fluoroscopy and tomography are conventionally used as guiding imaging modalities, ultrasonography has also been employed increasingly for this purpose in recent years [6,7].

Fluoroscopy guidance is widely accepted as the gold standard for caudal ESI [8,9]. The advantages of fluoroscopy include the easy visualization of the sacral hiatus, the ability to establish a reference point for the needle tip position within the sacral canal, and the capability to verify the accuracy of the injection by observing contrast material distribution after needle placement, ruling out intradural, intravascular, or contralateral distribution of the lesion. However, there is a significant radiation hazard for both the practitioner and the patient [10].

Ultrasonography have had a rapid increase in its popularity for caudal ESI with its advantages of no radiation exposure for both the patient and the physician, dynamic imaging capabilities, ease of repeatability, and potential for use without the need for operating room conditions [11,12]. With ultrasonography, the passage of the needle through the sacrococcygeal ligament and its placement into the canal can be monitored in real-time. However, the advancement of the needle within the sacral canal cannot be visualized due to the bone's interference. Hence, the confirmation of contrast distribution cannot be achieved with ultrasonography. Intravascular or intradural distribution of the medication cannot be reliably detected under ultrasound guidance. Therefore, even if the needle is visualized inside the sacral hiatus, the practitioner cannot be entirely certain about the accuracy of the caudal ESI.

Instead of using these two imaging methods separately, a hybrid technique may eliminate these drawbacks. With the hybrid technique, the amount of radiation exposure from fluoroscopy can be reduced. Besides, a safer and more accurate procedure can be ensured, which cannot be achieved using ultrasound alone.

The objective of this study is to evaluate the effect of caudal ESI under hybrid ultrasound/fluoroscopy guidance compared to the standard fluoroscopy-only method, focusing on procedure time and radiation exposure. The study hypotheses are as follows: (a) the hybrid technique will result in lower radiation exposure and shorter procedure time compared to the fluoroscopy-only method; (b) both techniques will achieve similar levels of improvement in patients with low back pain.

## 2 Methods

### 2.1 Study design and patients

A prospective randomized controlled study was conducted. Ethical approval was received from the local ethics committee (Approval number: E2-21-499). Clinicaltrials.gov database registration (NCT05145842) was made for this study. Ninety-seven patients with complaints of low back pain who presented to the Interventional Pain Unit of the University of Health Sciences Turkiye, Ankara Gaziler Physical Medicine and Rehabilitation Training and Research Hospital between May 2021 and May 2022 were assessed for the study. Inclusion criteria were (1) having radicular low back pain with evidence of nerve root compression due to herniated discopathy or spinal stenosis shown on magnetic resonance imaging, along with medical history, physical examination, and electromyography, (2) being over 18 years of age. The exclusion criteria for the study were as follows: (1) having cauda equina syndrome or progressive motor deficit, (2) having undergone spinal surgery previously, (3) presence of local infection or skin integrity impairment at the injection site, (4) history of allergy to contrast material or local anesthetics, (5) being in an acute or chronic unstable medical condition, (6) any cognitive or psychiatric condition that prevents understanding and completing the outcome measures, and (7) receiving anticoagulant therapy.

**Fig. 1:**
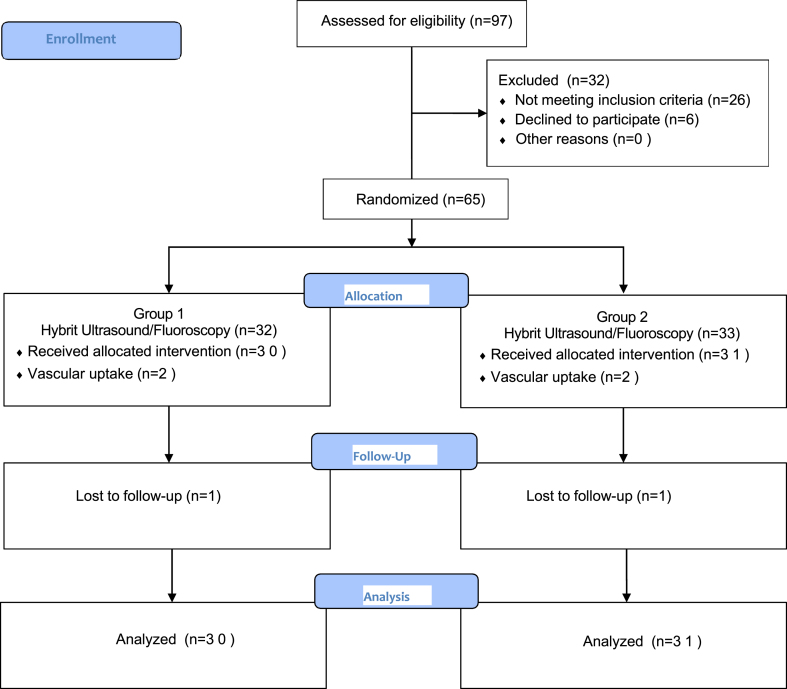
The study flow diagram.

Sixty-five patients who met the inclusion criteria were divided into two groups according to a computer-based randomization scheme. In the hybrid ultrasound/fluoroscopy group (Group 1), the procedure commenced with ultrasound guidance, followed by the injection under fluoroscopy guidance. In contrast, in the fluoroscopy group (Group 2), the caudal injection was performed exclusively under fluoroscopy guidance. Two patients in the Group 1 and teo patients in the Group 2 were excluded from the study due to the spread of contrast material into the vascular area during the procedure. Additionally, one patient from each group was excluded from the study for not attending the 2-week follow-up appointments. The study flow diagram is shown in Fig. 1. Written informed consent was obtained from all patients. All caudal ESIs were performed by a single physician with over ten years of experience in ultrasonography and fluoroscopy.

### 2.2 Ultrasound scanning

**Fig. 2:**
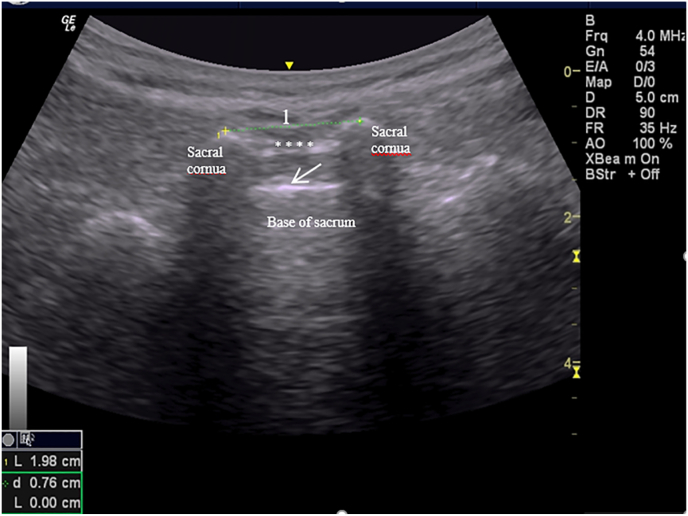
Width of the sacral hiatus. To demonstrate the caudal region, a transverse section is shown. Ultrasound reveals two sacral cornua as two hyperechoic reversed U-shaped structures. The arrow indicates the dorsal surface of the sacrum, while the asterisks correspond to the sacrococcygeal ligament. The distance between the tips of the sacral cornua was measured to determine the width of the sacral hiatus (1).

**Fig. 3:**
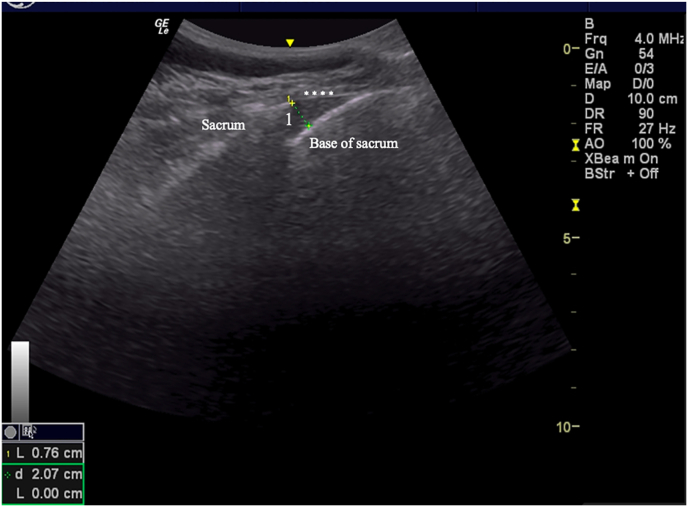
Diameter of the sacral hiatus. In the longitudinal section, the sacrococcygeal ligament, base of the sacrum, and the apex of the sacral hiatus were visualized. The asterisks correspond to the sacrococcygeal ligament. The distance between the sacrococcygeal ligament and the dorsal surface of the sacrum at the apex of the sacral hiatus was measured to record the diameter of the sacral hiatus (1).

Before the injection, all patients in Group 1 and Group 2 underwent ultrasound scanning [with a 5–12 MHz linear transducer (Logic e portable; GE Healthcare, Jiangsu, China)] to examine the caudal region morphology and to identify the patients who had an anatomical unsuitability for the procedure based on the size of the sacral hiatus [13]. Patients were assessed in the prone position. First, the ultrasound probe was placed on the caudal region in a transverse section to visualize the sacral hiatus. In the transverse section, the sacral hiatus, consisting of two hyperechoic sacral cornua in an inverted-U shape and the hyperechoic bands that bridge these structures from below and above, was identified. The lower band was the dorsal surface of the sacrum, while the upper band was the sacrococcygeal ligament. The distance between the tips of the sacral cornua was measured in the transverse view to determine the width of the sacral hiatus (Fig. 2). The ultrasound probe was then be rotated 90° to obtain a longitudinal view. In this view, the sacrococcygeal ligament, the dorsal surface of the sacrum, and the apex of the sacral hiatus were visualized. At the apex of the sacral hiatus, the distance between the sacrococcygeal ligament and the dorsal surface of the sacrum was measured and recorded as the diameter of the sacral hiatus (Fig. 3). Nobody had an anatomical unsuitability for the procedure.

### 2.3 Procedures

**Fig. 4:**
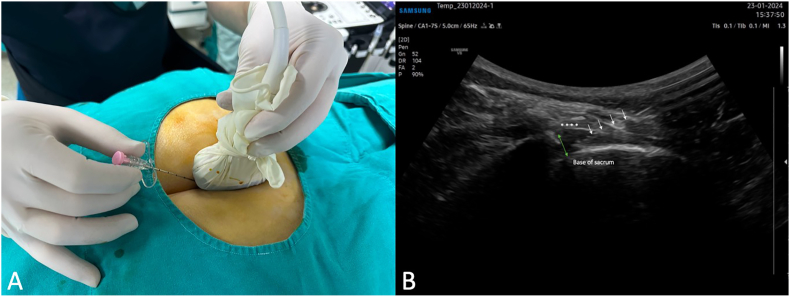
(A)Advancing the spinal needle in real-time ultrasound guidance. (B) Long axis view showing the needle inside the sacral canal. The asterisks correspond to the sacrococcygeal ligament. The arrows pointing to the needle. Two headed arrow pointing to the sacral hiatus.

**Fig. 5:**
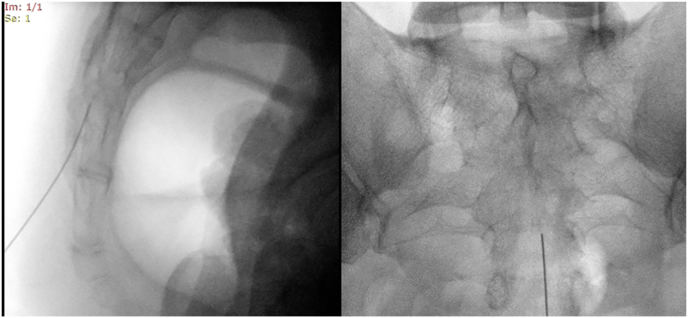
Advancing the spinal needle into the sacral canal was achieved by taking anteroposterior and lateral images.

After completing the ultrasonography scan, the procedures were performed with the patients in a prone position and the pelvis supported with a pillow from underneath. In the hybrid ultrasound/fluoroscopy group, the caudal region was first visualized in the longitudinal section under ultrasound. The spinal needle was advanced through the sacrococcygeal ligament under real-time ultrasound imaging (Fig. 4). Once the needle is within the sacral canal, no further advancement is made to ensure precise placement of the needle. The procedure will proceed to the fluoroscopic phase of hybrid guidance. Initially, a lateral view under fluoroscopy (Ziehm Vision FD Vario 3D, Ziehm Imaging GmbH, Nürnberg, Germany) was obtained to confirm that the needle tip was positioned within the sacral canal. Following that, the needle tip was repositioned just below the level of the third sacral vertebra (S3) in the anteroposterior view. In the Fluoroscopy group, the advancement of the needle into the sacral canal was guided by anteroposterior and lateral fluoroscopic images (Fig. 5). Similarly, the needle tip was positioned at the level of the third sacral vertebra (S3). Fluoroscopic imaging was performed intermittently, as needed, rather than continuously.

**Fig. 6:**
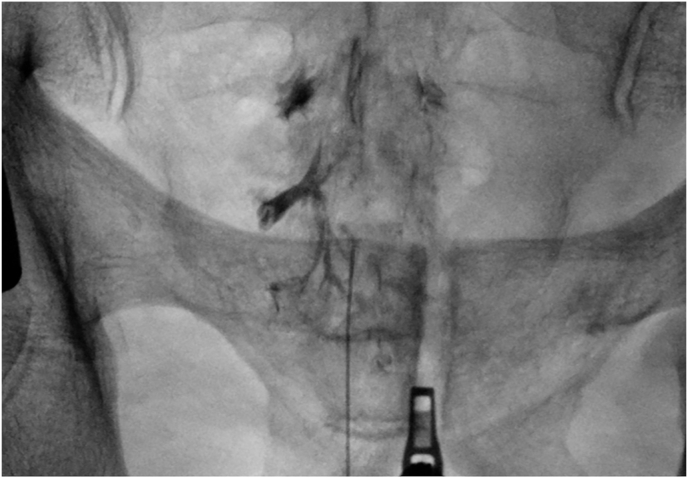
Christmas tree–like appearance after contrast injections.

An 3.5 inches 18-gauge Tuohy epidural spinal needle was used for the injection in both group. After cleaning the skin with povidone iodine, local anesthesia of the skin and subcutaneous tissues was achieved using 0.5 ml of 1 % lidocaine. After needle placement, 5 ml of contrast agent (Omnipaque 240; GE Healthcare, Princeton, NJ, USA) was injected to ensure the distribution of the contrast material appeared as the typical “Christmas tree–like appearance” pattern for caudal epidural injections (Fig. 6). A total of 8 ml of medication including 4 ml of dexamethasone at 4 mg/mL (a total of 16 mg dexamethasone), 2 ml of 1 % lidocaine, and 2 ml of saline was administered. The needle was removed, and the procedure was concluded. If an intravascular contrast flow was seen, the needle was removed without injecting any medication. The patients with intravascular uptake were excluded from the study.

### 2.4 Outcome measures

The primary outcome measure of the study was the procedure time (seconds). The time was measured using a stopwatch by a researcher present in the procedure room. The stopwatch was started when the needle tip of the local anesthetic syringe touched the skin and stopped when the spinal needle was withdrawn at the end of the procedure. Elapsed time of the needle insertion into sacral hiatus (T1) and elapsed time of the entire procedure (T2) were measured.

To assess radiation exposure, the parameters fluoroscopy time in seconds and kerma-area product (KAP), automatically measured by the fluoroscopy device, were used [10]. KAP is defined as a measure of the total x-ray energy leaving the x-ray tube and is measured in Gray-square centimeters (Gy·cm^2^). KAP is independent of the source-to-skin distance and when the radiation field is confined to the patient, it serves as a good measure of the total x-ray energy absorbed by the patient [14].

Patients' back pain intensity was evaluated using the Numeric Rating Scale (NRS) before the procedure and two weeks later. In this scale, the patient was asked to rate the pain level within the range of 0–10 cm (0 meaning no pain; 10 meaning the most severe pain) [15].

The level of disability related to pain for patients was evaluated using the Oswestry Disability Index (ODI) before the procedure and two weeks later. This index is divided into 10 subsections to assess the impact of pain on various physical activities such as sleep, personal care, social life, sexual life, and travel. Each subsection is scored between 0 and 5. The scores are summed up to obtain a score ranging from 0 to 50, with higher scores indicating increased levels of disability related to pain [16].

### 2.5 Statistical analysis

The sample size calculation via the G∗power (V3.1) program estimates that 32 patients per group, totaling 64 patients at least, will provide enough power (80 %) to detect a 10 % reduction in procedural time with the hybrid technique, accounting for a 20 % dropout rate [17].

SPSS Statistics v15.0 (SPSS Inc., Chicago, IL) was used for statistical analysis. A nonsignificant Shapiro–Wilk test showed the data distribution's normality. Continuous data were demonstrated as the mean ± standard deviation and median (interquartile range). Percentages were given for categorical data. Nominal data analysis was conducted using the Pearson χ2 test. Independent samples *t*-test was used for comparing two groups. Repeated-measure analysis of variance (ANOVA; two-way) was used for comparison of differences across different time points within groups and between groups, and post-hoc analysis was performed with the Bonferroni test. Statistical significance level was determined as *p* < 0.05.

## 3 Results

There were no significant differences between the groups regarding demographic characteristics, duration of pain, cause of low back pain, lesion level, width of the sacral hiatus, and diameter of the sacral hiatus. Additionally, baseline pain levels (NRS) and ODI scores showed no significant differences (p > 0.05) (Table 1).

**Table 1:** Patient characteristics.

	Group 1 (Hybrid Ultrasound/Fluoroscopy) (n = 29)	Group 2 (Fluoroscopy-only) (n = 30)	*p*
Age^a^ (years)	52.93 ± 15.21	57.96 ± 13.94	0.619
Sex			0.902
Female	14 (48.3 %)	14 (46.7 %)	
Male	15 (51.7 %)	16 (53.3 %)	
BMI^a^ (kg/m^2^)	27.74 ± 4.66	28.92 ± 4.61	0.922
Duration of Pain^a^ (Month)	8.41 ± 6.72	10.20 ± 1.10	0.143
Cause of Low Back Pain			0.920
Spinal stenosis	18 (62.1 %)	19(63.3 %)	
Lumbar disc herniation	11 (37.9 %)	11 (36.7 %)	
Level of Lesion			0.708
L4-L5	16 (55.2 %)	18 (60.00 %)	
L5-S1	13 (44.8 %)	12(40.0 %)	
Diameter of the sacral hiatus^a^ (cm)	0.44 ± 0.14	0.44 ± 0.09	0.134
Width of the sacral hiatus^a^ (cm)	1.66 ± 0.27	1.55 ± 0.45	0.582
Baseline ODI^a^	20.82 ± 9.71	23.66 ± 9.06	0.929
Baseline NRS^a^	5.48 ± 2.50	5.61 ± 2.35	0.972

BMI: Body Mass Index; NRS:Numeric Rating Scale; ODI: Oswestry Disability Index.

a Mean ± standard deviation.

The time of the spinal needle insertion into the sacral hiatus (T1) was statistically significantly shorter in the Group 1 compared to the Group 2 (p = 0.027). However, there was no statistically significant difference in entire procedure time (T2) between the groups (p = 0.100) (Table 2).

**Table 2:** Study outcome data.

	Group 1 (Hybrid Ultrasound/Fluoroscopy) (n = 29)	Group 2 (Fluoroscopy-only) (n = 30)	p
T1^a^ (min) (Time of the needle insertion into sacral hiatus)	2.82 ± 1.07	3.73 ± 2.47	0.027
T2^a^ (min) Entire procedure time	5.14 ± 1.55	5.86 ± 2.71	0.100
Fluoroscopy time^a^ (min)	0.06 ± 0.01	0.09 ± 0.03	0.010
KAP (cGy·cm^2^)^a^	43.73 ± 16.90	72.39 ± 32.75	0.026

Min: minutes; KAP: Kerma Area Product; cGy·cm^2^: Gray-square centimeters.

a Mean ± standard deviation.

Fluoroscopy time was significantly lower in the Group 1 compared to the group 2 (p = 0.001). Similarly, there was significantly decreased KAP (Gy·cm^2^) in the Group 1 compared to the Group 2 (Table 2).

The results of the repeated-measure analysis of variance are detailed in Table 3. A significant effect of time was observed for all outcome measures in both groups. Upon comparing the baseline NRS and ODI measurements with the measurements at 2 weeks post-injection, a significant improvement over time was noted for both groups (p < 0.001 for both). No significant interaction between group and time was identified concerning the NRS (p = 0.177) and ODI (p = 0.207) scores (Table 3).

**Table 3:** Study outcome data.

Outcome measures	Score		*Within Group Change*	*Time*	*Repeated Measure ANOVA F (p)*
	Baseline 2	Weeks	*Baseline vs 2 weeks*		*Time×Group*
ODI					
*Group 1* (Hybrid Ultrasound/Fluoroscopy)	21.0 ± 10 [12.0(20.0–28.0)]	17.9 ± 9.0 [12.5(18.0–21.0)]	−3.5 ± 3.0 [-2.0(-7.0-1.0)]	32.23 (<0.001)	0.29 (0.207)
*Group 2 (Fluoroscopy-only)*	23.3 ± 9.6 [17.5(20.0–29.7)]	17.6 ± 9.5 [11.0(16.0–21.0)]	−5.4 ± 7.5[-4.0(-10.0–1.0)]		
NRS					
*Group 1 (*Hybrid *Ultrasound/Fluoroscopy)*	5.2 ± 1.7[4.0(6.0–6.0)]	4.2 ± 1.8 [4.0(3.0–6.0)]	−1.0 ± 1.4 [-1.0(-2.0-0.0)]	23.22(<0.001)	1.54(0.177)
*Group 2 (Fluoroscopy-only)*	5.3 ± 1.8 [4.0(5.0–6.25)]	3.6 ± 1.9 [3.0(3.0–6.0)]	−1.8 ± 1.7 [-2.0(-3.0-1.0)]		

NRS: Numeric Rating Scale; ODI: Oswestry Disability Index.

∗Mean ± standard deviation [Median (25–75 quartile)].

ANOVA: Analysis of variance.

## 4 Discussion

In the present study, caudal epidural steroid injection (ESI) performed using hybrid ultrasound/fluoroscopy guidance showed significantly lower fluoroscopy time and KAP values compared to those performed with fluoroscopy guidance alone. Additionally, hybrid ultrasound/fluoroscopy guidance significantly reduced the time for needle entry into the sacral hiatus compared to fluoroscopy alone, although the reduction in the overall procedure time did not reach statistical significance.

It is known that error rates are high (20–38 %) in caudal epidural injections performed without the use of imaging techniques [5]. Therefore, it is recommended in today's practice those injections be performed with guided imaging. The advantages of fluoroscopy include easy visualization of the sacral hiatus, determination of the needle tip's limit point in the sacral canal (sacral 3), and the ability to assess the accuracy of the injection by observing intradural or intravascular distribution of the contrast material after needle placement [9,18]. However, fluoroscopy guidance poses a significant radiation risk for both the practitioner and the patient.

In ultrasound-guided procedures, there is no radiation exposure. The sacral hiatus, forming the entrance to the sacral canal, and the overlying sacrococcygeal ligament can be visualized in detail. The passage of the needle through the sacrococcygeal ligament and its placement from the sacral hiatus into the canal can be monitored in real-time. However, due to the shielding effect of bone tissue, it is not possible to visualize how far and in what direction the needle has advanced within the sacral canal and the distribution of contrast material [19,20].

In the literature, it is observed that caudal epidural injections performed under ultrasound and fluoroscopy guidance result in similar improvements in pain and functional scores [[21], [22], [23]]. In the study by Güler et al. [24] the position of the needle advanced under ultrasound guidance was confirmed using fluoroscopy and compared with the classical method of fluoroscopy guidance. Similar to our study, improvements in pain scores were observed in both groups, while radiation exposure was found to be lower in the ultrasound group. Gupta et al. [25] introduced “ultrafluoro guidance,” a combination of fluoroscopy and ultrasound, as a new technique for caudal injections. In a case series, Gupta et al. [26] performed caudal anesthesia with ultrafluoro guidance on four patients diagnosed with ankylosing spondylitis who had a history of multiple traumatic and unsuccessful subarachnoid blocks guided by blind and fluoroscopic methods due to ossification and syndesmophyte formation in the axial skeleton for perineal surgeries. Similarly, in our study, instead of using these two imaging methods separately, we used them as complementary methods. With this combination, we observed a decrease in the time for the needle to enter the sacral hiatus and the amount of radiation exposure.

Hazra et al. [27] conducted a study where the needle entry time into the sacral hiatus was found to be shorter in the ultrasound group compared to the fluoroscopy group (119 ± 7.66 vs. 222.28 ± 29.65 s, respectively, p = 0.0001). Similarly, in our study, the hybrid ultrasound/fluoroscopy guidance shortened the time for needle entry into the sacral hiatus compared to using fluoroscopy alone (2.82 ± 1.07 min and 3.73 ± 2.47 min, respectively, p = 0.027). There was no significant difference in the total procedure time between the groups. It is known that the experience of the performing physician is directly related to the procedure time [28,29]. In our study, the fact that all procedures were performed by a single physician with over 10 years of experience in fluoroscopy and epidural steroid injections could explain this situation.

Although fluoroscopy guidance is considered the gold standard for caudal epidural injections, exposure to radiation during the procedure is a significant concern [9,17,18]. The biological effects of radiation exposure are traditionally classified into stochastic effects and deterministic effects [30]. Stochastic injuries (cancer induction) arise from the mis repair of damage to the DNA of a single cell, resulting in a genetic transformation. There is no threshold value, and effects can be observed even at low doses. Deterministic injuries largely result from the unsuccessful attempt of cells to divide or differentiate due to radiation. There is usually a dose threshold, and the intensity of the effect varies according to the radiation dose [10,14,24].

If full compliance with safety measures is ensured, it has been stated that the radiation exposure is acceptable for both the patient and the doctor. However, it is known that especially for the practitioner, cumulative radiation exposure poses a significant long-term risk [10]. Cumulative radiation exposure is known to have adverse effects on the skin, gonads, and blood cells, increasing the risk of cataracts and cancer [31]. Therefore, the radiation dose should be minimized to the smallest dose necessary for adequate image quality and imaging guidance [29,32]. The principle of As Low As Reasonably Achievable (ALARA) has been supported by experts. The ALARA principle emphasizes the importance of short fluoroscopy time, low radiation dose, the use of pulsed fluoroscopy and collimation, distancing from the radiation source, and the appropriate use of shields such as aprons, thyroid protectors, gloves, and goggles [33]. In the present study, for all these reasons, we aimed to ensure the safest injection possible by reducing radiation exposure.

The kerma-area product (KAP), measured in Gy·cm^2^, quantifies the total X-ray energy emitted from the X-ray tube and serves as an effective measurement of the energy absorbed by the patient, thereby allowing for an estimation of the procedure's stochastic risk [14]. The current study, in Group 1, the fluoroscopy time was 0.06 ± 0.01 min, and the mean KAP level was 43.73 cGy cm^2^, while in Group 2, the fluoroscopy time was 0.09 ± 0.03 min, and the mean KAP level was 72.39 cGy cm^2^. In a retrospective study by Sacaklidir et al. [34], the fluoroscopy time for caudal steroid injection in 371 patients was 41.5 s, and the average radiation dose was reported as 0.218 mGy m^2^ (218 cGy cm^2^). In another study, the fluoroscopy time for caudal injection in 47 patients was 18.2 s, and the average radiation dose was reported as 101.8 μGy m^2^ (101.8 cGy cm^2^) [35]. The fluoroscopy times and mean KAP levels in these studies were higher than those in our study. The differences in fluoroscopy time in these studies could be attributed to variations in the experience of the physicians performing the procedures. In our study, all procedures were performed by a single physician with over 10 years of experience in fluoroscopy and epidural steroid injections. Additionally, differences in patient numbers could be another contributing factor.

During caudal epidural injections, intravascular injection has been reported in 3–14 % of cases, even after negative aspiration [36,37]. In our study, a total of 4 patients (6.1 %) out of 65 were excluded from the study due to intravascular uptake of the contrast material. The spread of contrast material into the intravascular space, which cannot be demonstrated solely with ultrasound guidance, were able to be visualized using fluoroscopy. It suggests that the hybrid guidance is more accurate than ultrasound-only guidance as well as safer than fluoroscopy-only guidance.

In caudal epidural injections, the sacral hiatus is a critical bony structure. It has been reported that a sacral hiatus diameter of <3.7 mm may be associated with difficulty in placing the needle into the epidural space [38]. Therefore, all patients were examined using ultrasound before the procedure to assess sacral hiatus morphology and identify any anatomical variations that could pose obstacles to the injection. No anatomical variations hindering needle placement were found in either group.

A significant reduction in NRS scores and ODI values at the second week compared to baseline was observed in both groups. Existing evidence, including the present study, supports the improvement in VAS and ODI scores using both fluoroscopy-guided and ultrasound-guided approaches [2,19,22,24,39].

A limitation of the study may be the low sample size which may cause a type 2 error meaning the study fails to detect a true difference when one actually exists. That may be reason of lack of significant difference in entire procedure time in the present study. The lack of specification of patients' Body Mass Index (BMI) may be noted as another limitation. It is known that the radiation dose and procedure time applied in fluoroscopy-guided spinal injections correlate positively with patients' BMI. Therefore, to obtain a more precise approach, radiation doses should be adjusted with BMI or doses per BMI should be provided for each procedure [34,40].

## 5 Conclusion

With the hybrid ultrasound/fluoroscopy guidance, a reduction in the time required for entry into the sacral hiatus and radiation exposure can be achieved. Additionally, this combination may mitigate the risk of intradural, and intravascular uptake compared to the use of ultrasound-alone. The hybrid guidance may offer a potentially safer method minimizing radiation risk compared to fluoroscopy-only guidance for caudal ESIs.

## Declaration of generative AI and AI-assisted technologies in the writing process

During the preparation of this work the author(s) used ChatGPT 4.0 in order to improve language. After using this tool/service, the author(s) reviewed and edited the content as needed and take(s) full responsibility for the content of the publication.

## Declaration of competing interest

The authors declare that they have no known competing financial interests or personal relationships that could have appeared to influence the work reported in this paper.
